# Reports on Hospitals of the United Kingdom

**Published:** 1914-04-18

**Authors:** Henry Burdett


### REPORTS ON

### Hospitals of the United Kingdom.

By SIR HENRY BURDETT, K.C.B., K.C.V.O.

### SERIES III.

### GENERAL KENT AND CANTERBURY HOSPITAL.

This hospital was opened for the reception of patients in 1793. Since then it has been enlarged, the floors of the wards have been relaid, new sanitary and other fittings have been placed in connection with several wards, and the remainder, we are informed, are to be modernised as soon as funds permit. This hospital has always and continuously laboured under the disability of a fixed revenue, which does not exhibit any elasticity, and of an expenditure which is some £700 more than the average income. This excessive expenditure is due to the ever-increasing demands for treatment. It is highly creditable to the managers, therefore, that although the buildings are old and, from several points of view, not up to date, they are maintained at such a high standard of efficiency.

The wards are roomy, though old-fashioned, provide an ample floor space per bed, and have some good points besides. Other portions of the ward units are not up to date, but attempts have been made, by the introduction of new fittings, and in other ways, to attain the highest possible efficiency. The linen and other cupboards are ample, and we found them in perfect order throughout.

There is no doubt that the patients in this hospital are comfortable, that the medical and surgical work is well and efficiently done, and that the institution renders good service to the community resident within its area.

### A Valuable Improvement.

The operation theatre is well equipped and efficiently lighted, but a difficulty must be experienced in heating it in the cold portions of each year owing to the large top light and the cold-air chamber which occupies the space immediately below it. We should recommend the closing in of this space by filling in the square opening below the top light and putting a radiator in the chamber thus formed immediately above and outside of the theatre proper. We were much struck by the presence of a mass of ironwork which stood in the middle of the female surgical ward and contained two large slow-combustion stoves consuming a very large amount of coal. These fireplaces are practically useless for heating purposes. The erection in question must have cost a very large sum, and in our view, whoever is responsible for the introduction of this faulty apparatus, and the makers of it, should be invited to remove this structure, not only from the female surgical, but from every other ward where it has been placed. It never ought to have been introduced into the wards of this hospital, which were formerly well and properly warmed by open fire-grates at the ends of each ward referred to, and we should hope, for hygienic reasons and for the sake of the comfort of the patients, that new fireplaces may be inserted at the end of each ward, and that the present practically useless and extravagant erection in the centre of the ward will be entirely removed. The failure of the apparatus in question causing an abundance of smoke to enter the wards, an absence of heating power which makes the wards undesirably cold and uncomfortable for the patients, and the excessive cost of the large consumption of coal, all combine to make it necessary that these erections should be abolished.

[Note.—We called attention to these matters in the visitors' book, and are glad to be able to report that the committee have adopted our suggestions.]

### The Need for Rebuilding.

Before many years are over, this hospital will have to be rebuilt upon a plan which will tend to reduce the annual upkeep and provide the committee with a modern, up-to-date, well-equipped, and carefully planned hospital. It would have been impossible for the present buildings to have continued so long in occupation for hospital use, where the most serious operations are successfully performed, had it not been for the excellence of the internal economy and administration under the direct supervision of the matron, Miss A. M. Messum, an old St. Thomas' Hospital sister, and one of the first nurses to join the Royal National Pension Fund for Nurses. For twenty-seven years Miss Messum has bravely and successfully borne the many trials and difficulties imposed upon her owing to the age of the existing buildings, the relatively small and insufficient income of the hospital, and the continual worries which necessarily arise in such circumstances. The spirit throughout the hospital is excellent, the patients were contented and light-hearted, especially in the children's ward, where they were very lively and extremely happy. One patient, a boy who had had a sharp attack of typhoid fever and had occupied a bed for eight weeks in the ward, was singing merrily to himself, and when asked the reason explained that he was to get up on the morrow.

Some of the wards have ample verandah accommodation, but it is not at present covered over so as to enable the patients continuously to sleep out in the open air. We understand that this requirement will be met very shortly in connection with one of the large wards, as a memorial to a member of the staff who did excellent service and was deservedly popular.

The hospital contains ninety-two beds, and exhibited one phase of the effect of the National Insurance Act upon hospital treatment which we have not met with so markedly elsewhere. There are a good many vacant beds on the male side of the hospital caused by the circumstance that relatively trivial ailments are not now admitted, as formerly, to in-patient treatment, because the sick benefit enables the patient to remain at home whilst the medical benefit pays the panel doctor for attendance on their needs and the chemist supplies them with medicine free of cost. Put another way, it shows that where hospital beds have been occupied by chronic and relatively trivial cases in the past, these beds will be free for more serious cases and are likely to be occupied by those cases, even at Canterbury, directly the Insurance Act has got completely to work, a system of adequate in-patient treatment has been set up, and everybody included under its provisions has been brought within the meshes of its organisation.

The out-patient department, which from the nature of the materials used in its construction is by no means an easy one to keep smart in appearance, was commendably well kept throughout. The out-patients pay a registration fee of 1s.

Finance.

The annual report of this hospital is a business-like document, and contains clearly-rendered accounts, including a balance sheet and financial statistics for the year and an excellent washing return, which reflect great credit on the treasurers and the secretary. We observe that though a tabular statement of the ordinary expenditure for the years from 1871-95 is included in the report, the table relating to the ordinary and extraordinary receipts is limited to the years 1896 to 1912. It is desirable that in future reports the statement should be extended so as to include the years 1871 to 1895, as is done in the case of expenditure. Each item of the ordinary income for the year 1912 reaches an average amount, but in the case of legacies, under the head of extraordinary receipts, the yield is at present infinitesimal, and far below the average for the years preceding 1905. Benefactions—that is, gifts of round sums and large donations—appear to have been *nil* during each year from 1908 to 1912. We would suggest that some active steps should be taken to make the many excellences of this hospital known to the wealthier residents throughout the district served by the hospital, when we have little doubt that both legacies and benefactions might be made to yield an annual sum, at least equal to the average of former years, during which periods these particular items seem to have been worked with considerable energy and success. The average expenditure for the three years ending 1912 amounted to £3,995—that is, to upwards of £300 less than the average for the previous three years.

This points to careful management, but we incline to think that the committee would be justified in taking to heart the proverb, "There is that spendeth and yet increaseth," and so to open their purse-strings sufficiently gradually to bring the upkeep of the whole institution to a higher state of efficiency than it exhibits at present. The more speedily and thoroughly the committee set to work to bring the equipment and ward units up to the standard of a modern hospital, the better position will they be in when the opportunity offers, as offer it must in the near future, to avail themselves of the changes which are pending in regard to the provision of in-patient treatment for insured persons, on payment of the "pro rata" cost to the hospital, plus 10 per cent. to defray the cost of the medical and surgical treatment. We urge this point because we are glad to note that the report deals fully and with wise intelligence with the effects which National Insurance is likely to have upon the position and work of this institution.

